# Development of Children in Iran: A Systematic Review and Meta-Analysis

**DOI:** 10.5539/gjhs.v8n8p145

**Published:** 2015-12-17

**Authors:** Firoozeh Sajedi, Mahbobeh ahmadi Doulabi, Roshanak Vameghi, Alireza Akbarzadeh Baghban, Mohammad Ali Mazaheri, Zohreh Mahmodi, Erfan Ghasemi

**Affiliations:** 1 Professor of Pediatrics; Pediatric Neurorehabilitation Research Center, University of Social Welfare & Rehabilitation Sciences, Tehran, Iran; 2 PhD Candidate of Pediatric Neurorehabilitation research Center, University of Social Welfare & Rehabilitation Sciences, Tehran, Iran; 3 Ph.D in Biostatistics, Associate Professor, Proteomics Research Center, Department of Basic Sciences, School of Rehabilitation Sciences, Shahid Beheshti University of Medical Sciences, Tehran, Iran; 4 Professor of Psychology, Shahid Beheshti University, Tehran, Iran; 5 PhD in Social Determinant of Health, Assistant Professor of Alborz University of Medical Sciences, Karaj, Iran; 6 PhD Candidate of Biostatistics Department of Biostatistics, Shahid Beheshti University of Medical Sciences, Tehran, Iran

**Keywords:** development, developmental delay, child, risk factors, prevalence, meta-analysis, Iran

## Abstract

**Background:**

In order to gain a better perspective of the developmental status of children in different regions of Iran, this study was carried out to determine the prevalence and the factors impacting child development in Iranian studies.

**Materials and Methods:**

Articles published in Iranian and international journals indexed in the SID, PubMed, Scopus and Magiran databases from 2001-2015 were systematically reviewed using standard and sensitive keywords. After evaluating the quality of 155 articles in the initial search, 26 articles were analyzed according to the inclusion criteria. After investigations, meta-analysis was done for six studies and the results were combined using Random Effects model, and the heterogeneity of studies was evaluated using the I^2^ index. Data analysis was performed using STATA version 11.2.

**Results:**

Eagger & Beggs tests, respectively with 0/273 & 0/260 did not confirm the probability of publication bias in the data, but heterogeneity in studies was confirmed (p<0/001). On such basis, the pooled prevalence of developmental disorder based on Random Effect model was calculated to be 0.146, CI (0/107-0/184). The prevalence of developmental disorders in children in the studies reviewed was reported between 7 to 22.4%. The most important risk factors were in SES (Socio Economic Status) and Prenatal, Perinatal, Neonatal &Child groups.

**Conclusion:**

More extensive studies and early intervention with respect to causes of developmental delay in children seems necessary.

## 1. Introduction

### 1.1 Description of the Condition

Childhood plays a vital role in human growth and development. The experiences of this period affect the entire life of individuals as well as society in a way that childhood growth is considered to be one of the important components of health throughout life. Therefore, brilliant future belongs to nations that invest on children. Lack of adequate care in the early years of life as the most important years of children’s life leads to delay in child growth and development. Hence, children will not be sufficiently prepared for starting school. The experiences of the early years of life will underpin the future life of every human. If the children’s needs and areas of development are appropriately addressed during this period, they will be healthier and will have greater thinking and reasoning ability and better social and emotional skills (Shonkoff et al., 2012; Vargas-Barón, 2005; Irwin et al., 2007; World Health Organization & Unicef, 2012).

### 1.2 Definition of Development and Developmental Delay

Development definition:

Development refers to those variations that a human being achieves during life in order to develop physically, mentally, verbally and socially. Such variations are affected by genetic factors inherited from parents as well as environmental factors, nutrition and social stimulants. (Karsimzadeh, 2005)

In general, children who acquire developmental abilities with delay and lack the developmental features and skills appropriate to their age in comparison with healthy children are considered children with developmental delay (Baker, 2001). Developmental criteria might have delay from birth or might have decreased after a relatively normal period. Acquired developmental criteria might gradually waste or they might temporarily or permanently disappear due to different diseases. (Vameghi, 2006) According to global estimates, at least 200 million children less than 5 years old fail to reach their developmental potential (Grantham-McGregor et al., 2007).

### 1.3 Prevalence of Developmental Delay

The prevalence of developmental disorders varies throughout the world and accounts for a considerable number even in developed countries. Approximately 15-18% of children in different communities suffer from disabilities in speech and learning as well as emotional-behavioral disorders, and 15% of children have serious psychosocial problems (Glascoe, 2000; Rydz et al., 2006). Even a rate of up to 30% has been reported in populations at risk, and the prevalence reported in Iran was 18.7% to 19.8% in several cities (Akbari et al., 2012; Soleymani et al., 2009; Soleymani et al., 2013; Torabi et al., 2012). Eight percent of pre-school children have developmental delay in one or more areas from birth until the age of 6. These findings suggest the importance of timely diagnosis and treatment of developmental delay (Tervo, 2006).

According to estimates of the Academy of Pediatrics, developmental disorders are among the most frequent problems of children and are a priority in America’s health care system (Vohr et al., 2003). This rate is 15-20% in the United States, 15% in Jamaica, 8% in Bangladesh, 15% in Pakistan, 1.5-2.5% in children under 2 years in India, up to 10% in Iraq, 3.3% in Brazil, and 12.5% in the Netherlands (Al-Naddawi, & Alwan, 2013; de Moura et al., 2010; Poon et al., 2010; Potijk et al., 2013; Vohr et al., 2003). The prevalence of different aspects of developmental delay, including gross motor, fine motor, problem-solving, communication and social-personal domains has been reported as 3.87%, 4.04%, 4.31%, 4.15%, and 3.69%, respectively (Sajedi et al., 2012).

Unfavorable life conditions of pregnant mothers and their consequences can induce changes to the fetus environment and produce harmful effects on child’s development and physical and mental health (Maccari et al., 2003; Weinstock, 2001).

### 1.4 Risk Factors of Developmental Delay

The main cause of developmental delay remains unknown, but possible factors include biological, pregnancy, and environmental factors (Persha et al., 2007; Al-Naddawi et al., 2013). In many cases, no single, specific factor can be clearly identified as a reason (Persha et al., 2007). A wide range of social causes and factors contribute to its emergence. Development of children is affected by various hereditary, biological, psychosocial, and environmental factors. In other words, human development is a result of dynamic, continuous interaction between biological and acquired factors (de Moura et al., 2010). There is a widespread agreement that neither nature nor nurture alone determine the development process independently. It is rather determined by “nature through nurture” (Fernald et al., 2009).

Risks that threaten children’s and even adults’ health sometimes start from the fetal period (Soleimani et al., 2001). Infants with a history of one or more risk factors in the prenatal, natal, or postnatal periods are at risk for developmental delays (Sajedi et al., 2005). Family and its health conditions have a strong impact on early development of children, and any chronic physical or mental problem, violence, depression, and chronic diseases of mother (or primary caregiver) can adversely affect children (Irwin et al., 2007). Various studies have been done on factors affecting developmental delay in children (Glasson et al., 2004; Squires, Carter, & Kaplan, 2003).

Low levels of parental education, maternal age, low birth weight, preterm labor, and low maternal age at birth were considered an important factors affecting child development (Ryan-Krausetal, 2009; Hediger et al., 2002).

In some studies conducted in Iran, the factors such as diabetes, pregnancy, hypertension, familial marriage, history of abortion, high risk pregnancy, and low birth weight have resulted in developmental delay of children (Soleymani, Khoushbin, & Shams, 2001; Kousarian et al., 2007; Sajedy, 2005).

The importance of screening, diagnosis of infants and children at risk, and prevention of the consequences caused by developmental delays is very high, given the importance of children development and its widespread factors as the future makers of the country. Accordingly, identifying the prevalence of developmental delay across the country and its risk factors appears necessary. In this field, starting interventions prior to occurrence or progress of problem seems the most reasonable solution.

### 1.5 Why Is It Important to Do This Review?

Whereas children are the builders of future, and developmental delay are general problems for any country including Iran, summing up the results of studies in recent years can be important for a better identification of effective factors in developmental delay given the wide range of causing factors.

Since there are no organized reports about developmental delay in Iran, and different methods and tools are used in studies that have been done in order to diagnose the developmental delay, different results in the field of prevalence and causes are reported as well. Moreover, there are no Systematic Reviews and Meta-Analysis studies in this field to review all studies done in the country in recent decades and reach a quantity result.

The present study was thus carried out to systematically review the prevalence of developmental delay in children and find their main causes in Iran.

## 2. Methodology

Criteria for article selection and quality evaluation.

### 2.1 Search Strategy

This is a 14-year (2001-2015) systematic review of articles (in Persian and English) published on the prevalence of developmental delay and factors impacting it in Iran. The study began according to the standardized keywords of MESH in 4 domestic and foreign databases including SID (www.sid.ir), Magiran (www.magiran.com), PubMed, and Scopus using AND/OR operators.

### 2.2 Keywords

“child development” AND Iran-(develop AND child AND Iran)-”Development delay” AND Iran-(developmental AND children AND Iran)-(development AND children AND IRAN)-(develop AND children AND Iran)--(“developmental delay” AND children AND Iran)-(“Developmental delay” AND Risk factor AND Iran)-(“developmental delay “AND Risk factor OR “developmental abnormality “AND Iran)-(“developmental delay” AND prevalence AND Iran)

At first, a list of titles and abstracts of all articles searched in domestic databases was prepared and evaluated by two researchers. The articles with duplicate titles were excluded since they were printed both in Persian and English.

### 2.3 Quality Assessment

Then, the full text of articles was studied using the critical appraisal checklist (CACL) of STROB (Strengthening the Reporting of Observational Studies in Epidemiology) in order to find appropriate studies. The STROBE statement is a checklist of items that should be addressed in articles reporting on the three main study designs of analytical epidemiology: cohort, case-control, and cross sectional studies.

The checklist has 22 parts, each of which covers parts of an observational report and is awarded a score. These items relate to the article’s title and abstract (item 1), the introduction (items 2 and 3), methods (items 4-12), results (items 13-17), discussion sections (items 18-21), and other information (item 22 on funding). Eighteen items are common to all three designs, while four (items 6, 12, 14, and 15) are design specific (Von Elm et al., 2007).

If researchers did not agree on selecting a certain paper, a third expert judged the case. Regarding foreign databases, the process was similar to domestic databases. In one case which was no access to the full text article, it was requested from the corresponding author by email, and received via the same way.

### 2.4 Inclusion Criteria

1. All observational studies (cross-sectional, case-control and cohort).
2. Studies on the prevalence of developmental delay in children up to 8 years old (early child development: Early childhood is defined as the period from prenatal development to eight years of age. (Irwin et al., 2007)
3. Studies on factors influencing developmental delays in children.
4. Studies on any field of developmental delays [motor (gross motor, fine motor), problem solving, communication, and personal-social].
5. Studies of the last 14 years (2001-2015) in Iran.
6. Studies in English or Farsi.

### 2.5 Exclusion Criteria

1. Studies on high risk children, admitted to NICU (Neonatal Intensive Care Unit), LBW (Low Birth Weight) preterm labor (Gestational age <37 Week), *etc*.
2. Studies which related developmental disorder to genetic factors such as Cerebral Palsy (CP) and autism.
3. Studies irrelevant to the study plan (qualitative studies, studies of specific groups such as low birth weight infants, postpartum thyroiditis in mother, children with early treated phenylketonuria, low birth weight neonates, congenital hypothyroidism, infrequent voiding, etc…), interventional studies, tool development & standardization).
4. Any study on development that did not address prevalence and factors influencing developmental delay.
5. Acquiring the complete scores was necessary in methods, sampling, results and discussion parts in STROB checklist.

## 3. Results

**Figure 1:**
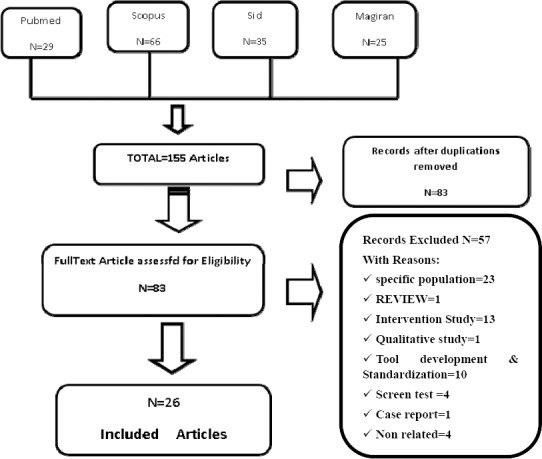
Selection flowchart of studies included in the review

The investigation conducted in 4 databases using the keywords resulted in 155 articles. Out of these articles, 4 articles were published in two separate journals or in two different languages. Owing to different reasons (Figure 1), 57 articles were excluded. Finally, 26 articles all of which remained in the final review given their minimum score of 15 out of 22 (Table 1) were evaluated according to STROB checklist by two researchers. Out of the final 26 papers, the methodology of 23 studies was descriptive (cross-sectional, analytical, correlational, comparative), 2 were case-control, and one was retrospective.

**Table 1:** Summary of studies on the prevalence and risk factors associated with developmental delay in studies conducted in Iran from 2001 to 2015

Author, Year City	Study type &Tool & AGE	N	Prevalence Delay Development	Risk factor	SCOR
Khabbazkar et al. (2015). Rasht	Descriptive-cross-sectional-ASQ 8-12 months	400 pairs of mother and child	14% in total Highest delay in the problem-solving area 13% and lowest in fine motor 5.8%	Domestic violence	22
Ali Akbari et al. (2012). Esfahan	Descriptive-cross-sectional-ASQ 4-60 months	401 pairs of mother and child	18.7% in total Highest delay in fine motor 7.2% and lowest in persona;-social 1.7%	Multiple pregnancy-LBW-Gestational diabetes-Recurrent abortion-Pregnancy disorders-Infant age	20
Kosarian et al. (2006)-Sari	Descriptive-cross-sectional-PEDS 4.28±1.31 years	736 children	-	Significant association existed between parents concern with parents education, residence, and history of child disease (*p*<0.5)	21
Ghahramani et al. (2002)-Gonabad	Descriptive-cross-sectional-Developmental indices of children questionnaire (gross motor, fine motor, language, and social communications)- 12 months	110 in two groups of rural and urban	Lowest frequency in gross motor: Child standing for varied time In fine motor: eating with spoon 67% In speaking and communication index: cooperation in wearing cloth 83%	Mothers age and occupation, preterm labor	18
Torabi et al. (2012)-Esfahan	Descriptive-cross-sectional-ASQ 4-60 months	401 pairs of mother and child	18.7% in total Highest delay in fine motor 7.2% and lowest in persona;-social 1.7% 95% CI (0.5-2.9)	LBW	20
Fallah et al.-(2013)-Yazd	Comparative-ASQ 60 months	122 children in two normal and assistive reproduction groups	Fine motor 47.5% vs. 24.6%-(p=0.008) and problem solving (60.6% vs. 34.4%, p=0.004) were more in ART born children	Mother education-birth weight	21
Nooh Jah et al., (2014). Dezful	Descriptive-analytic, researcher-made questionnaire including 20 items based on the checklist and criteria of WHO, the study was designed with six assessment indices of motor development 1-5 years, mean age = 26.3±14.6 months	800 children	12.4% of children had delayed motor development; the highest prevalence of delay in percentage 90 and 97 regarding walking age without help was 12% and 4%, respectively.	Gestational age, preterm labor	21
Soleimani et al. (2013). Qazvin	Descriptive-correlational-ASQ 12 months	250 mothers and children	Prevalence of delayed development 22.4%; the highest prevalence of delay in the area of communication 8% and the lowest in the area of problem solving 2%; 4.8% developmental delay in children of anemic mothers; 17.6% developmental delay in non-anemic mothers	Anemia had significant correlation (*p*<0.05) ----- Hemoglobin had significant correlation with developmental status of child (OR=0.31; *p*=0.02) Male gender was close to significance level (OR=0.55; *p*=0.55)	20
Soleimani et al. (2001). Karaj	Descriptive- INFANIB- 4 months	6150	Prevalence of developmental delay 8 per 1000	Infant: infection, CNS seizure, surgery, general anesthesia, neonatal convulsion, immaturity, septicemia, LBW Mother: disease history during pregnancy, history of abortion	19
Soleimani et al. (2010). Karaj	Descriptive- INFANIB 4-18 months	6150	-	Neonatal and postnatal seizures; preterm birth; LBW; type II pneumonia; pregnancy-complications (preeclampsia, gestational diabetes, Vaginal bleeding, X-ray exposure, and cervical incompetency); and history of miscarriage.	19
Soleimani et al. (2013). Karaj	Retrospective-INFANIB 4-18 months	1232 pairs of mother and child, Of 6150 subjects in two groups with and without developmental delay	-	LBW, preterm labor, sex, mothers problems during pregnancy, abortion, pneumonia type 2, neonatal convulsion, postnatal convulsion, asphyxia (neonatal or perinatal), parental consanguinity, type of delivery, maternal age	21
Soleimani et al. (2005). Karaj	Descriptive Analytic-INFANIB 0-18 months	6150 children	-	Immaturity, mothers problems during pregnancy, history of abortion, weight less than 2500 g, type 2 pneumonia, CNS disorder, neonatal convulsion, meningitis, encephalitis	19
Safari et al. (2009). Yasuj	Descriptive- Developmental indices with interview with mother 9.4±5.5 months	225	-	--	21
Dalvand et al. (2008). Tehran	Descriptive cross-sectional-PDMS 72-83 months	180 first school children	-	Mean development of fine motor and mean age are significant with total motor	20
Karami et al., (2015). Khorramabad	Descriptive cross-sectional-ASQ 12 months	764 children	9.7% of all areas of development delay t and 16.3% in at least one abnormal area	Birth weight, neonatal specific complications, high maternal age, pregnancy complications, epilepsy, smoking and alcohol consumption, family history of mental retardation, SES	20
Shah ahammadi et al. (2015). Pakdasht Varamin	Descriptive-cross-sectional-ASQ 4-12 months	210 children	Highest prevalence of development delay in personal-social area 8.6% Lowest disorder in communication area 3.8%	Mother’s education, mother’s occupation, child age, child nutrition	20
Abri et al. (2011). Varamin	Comparative-analytic 17-item Vineland social maturity scale 3-6 years	558 children	-	Children in kindergarten had higher social development that those without this service	18
Amouzadeh Khalili et al. (2004). Semnan	Descriptive-comparative Six criteria of assessment for identifying difference in performance of rural and urban children Mean age of urban children 3.63 years and mean age of rural children 3.82 years	97 children in rural and urban kindergarten	-	Being rural or urban child	21
Mohammadi et al. (2008). Sanandaj	Descriptive-comparative BMAT test Basic motor ability test 5-6 years	140 children in two groups of with and without presence in pre-school	-	No significant difference between boys in both groups in terms of basic skills development	18
Rahmani Rassa et al. (2014). Hamadan	Descriptive-analytic- PDMS 0-2 years	123 infants	-	Child age	21
Afraz et al. (2015). Yasuj	Descriptive-analytic-ASQ 4-12 months	200 mothers and children	7% speaking delay	Child weight	21
Sajedi et al. (2009). Tehran	Descriptive-analytic PEDS 1 month to 3 years	7500 children	18.7% per 1000 children	Prematurity (25.6%), low birth weight (19.2%), neonatal seizures (7.5%), hyaline membrane disease (6.7%), systemic infections of mothers during Pregnancy (5.9%), severe neonatal hyperbilirubinemia 5%,	22
Soleimani et al. (2014). Alborz	Descriptive-analytic INFANIB- 0-18 months Mean=10±4month	6150	20% in gross domain 25% in fine domain	-	21
Yaghini et al. (2012). Esfahan	Descriptive study-ASQ In 6 months old and re-examination at 24 months	800	10.5% at least in one area in 6 months, of these 84 children, 19.2% had problem in the communication domain, 30.7% in gross motor, 53.8% in fine motor, 26.9% in problem solving and 7.6% in personal social At 24 months, 38.4% Of them remained delayed; 21.1% in one domain, 9.6% in two domains, 3.8% in four domains and 3.8% in five domains. Of the children who had problem in communication, 20%; in gross motor, 25%; in fine motor, 20%; in problem solving, 30% remained delayed At 24 months	-	18
Khalaji et al. (2014). Arak	Descriptive-cross-sectional Gross motor development Ulrich test 4 and 5 years 8 and 9 years	60	-	Cousin marriage Significant effect of cousin marriage on manipulation skills and gross motor Significant effect on 8 and 9 years girls	20
Kordi. (2014). Tehran	Descriptive-correlational and cross-sectional-Denver2 3 to 6 years	206 children (113 boys and 93 girls)	-	Child age Child anthropometric characteristics	21

In terms of the tools used, the majority of studies (9) have used ASQ (Ages and Stages Questionnaire). 5 studies used INFANIB (Infant Neurological International Battery) and 2 studies used PEDS (Parents Evaluation of Developmental Status) tools.

One study used gross motor development Ulrich test, second edition and one study used BMAT (Basic Motor Ability Test). Among other studies, one used 17-item Vineland social maturity scale, one the children development indices questionnaire, one a researcher-made questionnaire, one the six-criterion assessment questionnaire for determining functional differences between rural and urban children, and one the developmental indices questionnaire in addition to interviews with mothers. (Table 1)

In order to perform meta-analysis in the second stage, the studies that used ASQ tool were included in the analysis. Finally, six of nine relevant studies were examined. One study was excluded because of same findings of another study with different title. Two studies were also excluded due to lack of estimation of overall prevalence of developmental delay and only mentioning developmental delay in some developmental domains (Table 2).

**Table 2:** Profile of articles examined in the meta-analysis on the developmental disorder in children in Iran

Study	ES	95% Conf.Interval		% Weight
Karami (2015)	0.163	0.137	0.189	17.72
Yaghini (2012)	0.105	0.085	0.125	18.31
Afraz (2015)	0.070	0.035	0.105	16.61
Khabbazkar (2015)	0.140	0.106	0.174	16.78
Soleimani (2013)	0.224	0.172	0.276	14.34
AliAkbari (2012)	0.187	0.149	0.225	16.24
D+L pooled ES	0.146	0.107	0.184	100.00

Heterogeneity chi-squared = 44.21 (d.f. = 5) p = 0.000; I-squared (variation in ES attributable to heterogeneity) = 88.7%; Estimate of between-study variance Tau-squared = 0.0020; Test of ES=0: z= 7.40; p = 0.000.

The highest prevalence of developmental disorder was reported in the study of Soleymani et al. as 22.4% (2012) in the city of Qazvin, and the lowest prevalence in the study of Afraz et al. in Kohgiluyeh and Boyer-Ahmad province as 7% (2015). For Publication bias, the Egger & Bagg tests were used and the P-value was calculated 0.273 & 0.260, respectively, indicating there is no publication bias in studies.

According to the I^2^ index which is used to verify the heterogeneity of studies, the heterogeneity diagnosis was confirmed in studies (I^2^ = 88.7%), (p<0.001). According to the Random Effects model, the Der simonian and Laird method was used and the Pooled prevalence was calculated 0.146 in the confidence interval of (0.107-0.184).

**Figure 2:**
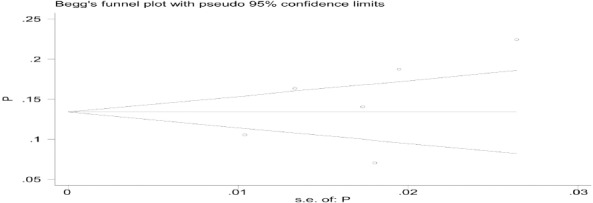
Shows forest plot based on the random effect model

Figure 2 shows Forest plot based on the random effect model.

**Figure 3:**
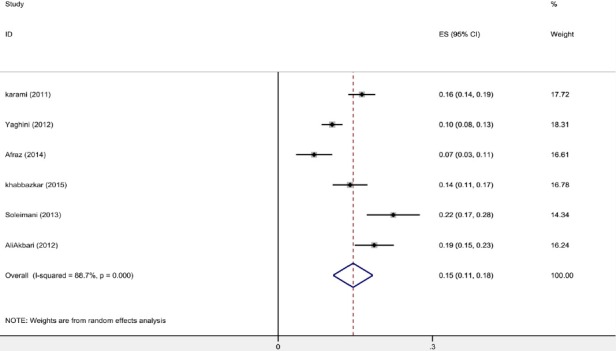
The prevalence of developmental disorder in children and its 95% confidence level in the examined studies in the year and city of the study based on the random effects model. The midpoint of each segment shows the prevalence amount and the segment length shows the 95% confidence level in each study. The diamond symbol indicates the total prevalence for all studies

Among the factors associated with developmental disorders in children, 26.1% were categorized in prenatal factors (history of mother’s previous or current diseases, abortion and infertility, parity, previous history of disability in children, multiple pregnancies, *etc*.), 21.65% in perinatal risk factors (maternal disease, obstetric factors, fetal factors, delivery type and place, use of illegal drugs, improper health behaviors, *etc*.), 28.67% in neonatal and child risk factors (diseases of infants and children and demographic factors), and 23.57% in SES group (parental education, family size, nationality, place of residence, type of housing, and occupation of parents) (Table 3).

**Table 3:** Frequency of studied characteristics on children-related studies

Variable		Frequency	Percent	Relationship		Non-relationship	

Prevalence							
Prenatal Risk Factors	History of maternal disease (epilepsy, gestational diabetes, asthma, gastrointestinal disease, anemia, radiotherapy, chronic disease)	9	100	5	55.6	4	44.4
	consanguineous marriage Cousin marriage	6	100	1	16.6	5	83.3
	Obstetrics history (infertility, abortion, delivery intervals, number of pregnancies)	11	100	4	36.4	7	63.6
	Dimension of family	1	100	-	-	1	100
	Maternal age	8	100	3	37.5	5	62.5
	Number of children	2	100	1	50	1	50
	History of developmental delay in previous children	1	100	-	-	1	100
	History of disability in previous children	3	100	1	33.3	2	77.7
Perinatal Risk Factors	Obstetrics problems (anemia, pregnancy age: preterm and post term labor, vaginal bleeding, bacterial infection, cx failure, hypertension, surgery, violence, pregnancy period diseases)	20	100	16	80	4	20
	Smoking and alcohol consumption	2	100	1	50	1	50
	Drug consumption	3	100	-	-	3	100
	Number of pregnancies and rank of delivery	5	100	1	20	4	80
	Multiple pregnancy	2	100	2	100	-	-
	Fetal factors (fetal distress and nuchal cord)	2	100	-	-	2	100
SES Risk Factors	Parents education	17	100	9	53	8	47
	Residence	5	100	5	100	-	-
	Housing type	1	100	-	-	1	100
	Nationality	1	100	-	-	1	100
	Economic status	6	100	4	50	2	50
	Parents occupation	11	100	3	27.3	8	72.7
	Child nutrition	4	100	1	25	3	75
Neonatal &child factors	Child age	6	100	4	66.7	2	33.4
	History of neonatal disease (neonatal convulsion, hyper bili, CNS infection, type 2 pneumonia, hyaline membrane disease, neonatal sepsis, exchange transfusion, neonatal anomaly, congenital heart disease)	10	100	8	80	2	20
	History of child’s disease (urinary sepsis, metabolic disease)	3	100	1	33.3	2	66.7
	Neonatal anthropometric factors) height, weight, head circumference, and Apgar)	9	100	3	33.3	6	66.7
	Child gender	9	100	4	44.4	5	55.6

## 4. Discussion

In this study, the prevalence of developmental delay was estimated 14.6 percent. Since the same sample size in all studies is used in the meta-analysis, it has high power and higher reliability, and gives more accurate estimates than other estimates conducted in different countries (Noble, 2006; & Cohn Becker, 2003; Cohn & Becker, 2003).

Given the importance of developmental delay, various studies compared the prevalence of developmental delay in the world. Accordingly, this study tried to examine the prevalence of developmental delay in Iran using meta-analysis in order to be effective in planning and timely interventions and providing services for children.

As mentioned in the results, only 6 cases among the studies reviewed were suitable for meta-analysis. Of heterogeneity causes of the studies, one can point to the difference in target groups, age range of selected children; Afraz (4-24 months), Ail akbary (4-60 months), khabazkar (8-12 months), and Yaghini (6 &24 months) only one age level such as 12 months were considered (Soleimani & Karami, 2015).

Other causes were the research environment (different places in the country) and differences in sample size (at least 200 cases in the study of Afraz et al, and 800 cases in the study of Yaghini et al.). The heterogeneity test confirmed these differences. Difference in the sample size of each study is one of the strengths or weaknesses of the study.

The difference in the population of mothers; between 26 revised studies, only Afraz et al. (2015) article overviewed teenage mothers (less than 19 years) while in other studies, mothers between 20-30 years were considered. Also in the study by Fallah et al. (2013), infants of normal pregnancy were compared with infants born by assisted reproductive techniques.

As mentioned above, the prevalence of developmental delay in this study was obtained 14/6% which is consistent with the results of Khabbazkar et al. studies in Rasht (2015) which reported the prevalence of developmental delay as14% in women with domestic violence and Karami et al. study in Khorramabad city which reported it as 16%. But they are inconsistent with results of Yaghini et al. studies in Isfahan (2012) which reported the prevalence of developmental delay in children referring for vaccination as 10.5% and Ali Akbari et al., (2012) in the same city which reported the prevalence of developmental delay in high risk pregnancies as 18.7%.

The highest reported developmental delay was reported for Qazvin (22.4%) in a study entitled “Relationship between maternal anemia during pregnancy with developmental delay, (2013) and the lowest prevalence (7%) for Afraz study of teenage mothers (under 19 years old during pregnancy) in Yasouj (2015).

Among factors associated with developmental delay in systematic review, the most frequently studied ones were as follows in descending order: neonatal, childhood, motherhood, and SES. The majority of neonatal studies did not find a significant relationship between anthropometric factors, neonate gender, and medical history of the child with developmental delays. These findings are compatible with results of studies in other countries. For instance, in some studies (Piek et al., 2008; Glasson et al., 2004; Bernard et al., 2013), no significant relationship was found between gender, anthropometric factors, and developmental delay in children. Only 33.3% of the investigated articles reported an effective, significant relationship between the mentioned factors and child’s developmental delay.

The inconsistencies in the results of studies in Iran may be caused by difference in the time interval of samples studied. This is a very effective factor, because in some articles, the time of study was from birth or with respect to the delivery case of mother. The other probable causes were in sample sizes and different sampling methods. Some of the studies were utilized randomized and some convenient sampling, but Ghahramani et al., (2002) and Safari et al., (2009) used census sampling method (Afraz et al., 2015; Ghahramani et al., 2002; Safari et al., 2009; Torabi et al., 2012). These differences in sampling method can affect the researcher’s bias.

Among maternal factors examined, the majority of studies reported a significant relationship with developmental delay and only 20% of the articles did not report a significant relationship. (Safari et al., 2009; Mohammadi et al., 2008; Rassa et al., 2014; Dalvand et al., 2008; Yaghini et al., 2012; Abri et al., 2011) This can be due to low-volume samples. For example, the sample size was 6000 in the study carried out by Soleymani et al. (2008, 2009, 2013), and 7500 in the study done by Sajedi et al. (2009). According to experts, the probability of finding a significant relationship increases by higher sample size (Munro, 2005).

Maternal factors associated with developmental delay can be noted as domestic violence, anemia during pregnancy, high-risk pregnancies and maternal age.

There are reports about domestic violence with developmental delay; domestic violence can indirectly affect children’s health through pregnancy problems and poor prenatal care and preterm delivery. It also causes emotional and physical inability of abused mothers, and mother cannot meet basic needs of her child (Huth et al., 2002; Kaye et al., 2006; Ziaei et al., 2014). A wide range of mechanisms may explain the relationship between domestic violence and health of the mother and child. For example, stress plays an important role among behavioral or biological factors (Silverman et al., 2006) and the effects of stress are reflected on the abused mother’s behavior and cause negative consequences for her pregnancy (Silverman et al., 2006; Kouba et al., 2005). Moses et al. found that mothers who experienced violence are at the risk of depression and confirmed the deleterious effects of depression on the infant development (2004).

Also on relation of maternal anemia with developmental delay, it is mentioned that fetus cells need iron for development of nervous system. The most relevant effect of iron is on laxative properties, the performance of menstrual complexes and decrease in neural-metabolic activity (Lozoff & Georgieff, 2006; Shafir et al., 2008; Corapci et al., 2006). Perez et al. (2005) found that maternal anemia is effective on developmental delay in the communication domain.

Also maternal age is another factor associated with child’s developmental outcomes. Teenage mothers are not mentally prepared to accept maternal feeling or lack parenting skills, which can disrupt the child’s development (Leigh & Gong, 2010).

Studies showed that mothers under 20 and over 35 years old deliver more infants with weighing less than normal compared to mothers between 20 and 29 years old, which is directly related to developmental disorder (Bae et al., 2011).

The socioeconomic status of parents is one component of infant health (Berghella et al., 2007). Studies show that the socioeconomic status of family has a significant negative correlation with fine motor function, IQ scale, verbal perception index, comprehensive analysis index, and working memory index (Piek et al., 2008). The nutritional interventions in infancy, which are associated with family economic status, can have a positive effect on growth and development of infants and children (Persha et al., 2007; Behzadnia et al, 2005).

In good financial status, it is also possible to create a favorable physical space for learning and stimulation of learning in children as well as buying better equipment at home environment and providing better services, which have positive impacts on children’s development (Propper & Rigg, 2007).

According to the studies, it seems that the socioeconomic status has a complex structure and it is unlikely that poor socioeconomic status is a direct, independent factor for developmental disorder of children. However, it might affect development by creating unhygienic behavior, more exposure to stress and psychological reactions to stress (Ceballo & McLoyd, 2002).

Effects of developmental disabilities affect both child’s family and society. Evidence shows that early treatment of developmental disorders leads to improvement of child outcomes and reduction of society costs (Poon et al., 2010).

Several studies have shown short-term and long-term benefits of early intervention in developmental disorders of children in terms of individual, family, economic, and social aspects, and early identification, treatment, and rehabilitation will be accompanied with better results in children. In addition to identification of children with developmental delays and treatment, intervention and training programs in healthy children such as cognitive-motional training can also improve the developmental outcomes (Miller & Clark, 1998; Miller & Clark, 1998; Sajedi & Barati, 2014).

## 5. Limitations

Among restrictions of the studies, one can point to different age ranges and similar study environments. For example, in the majority of studies, the samples were gathered from health centers, which can prevent its generalization to the whole country. Another restriction is that samples were not collected completely randomly and six studies were included in the Meta-analysis a limitation of the study.

## 6. Conclusion

In this review article, the results show that children’s developmental delay is a prevalent problem despite differences in sample size, sampling methods, tools used, etc. Several factors including maternal and neonatal factors are associated with the problem. Therefore, considering the articles’ results, it seems that timely, appropriate actions can reduce and control children’s developmental delay in many cases.

This study can help the health policy makers in direction to arrange suitable plans in order to prevent developmental delay in children with screening and on-time and necessary intervention.
